# Integrated multichannel seismic profiler dataset from the southeastern apulia continental margin (Otranto Channel, Southern Adriatic Sea): Insights into fault-controlled gas seeps and geo-hazards

**DOI:** 10.1016/j.dib.2026.112832

**Published:** 2026-05-07

**Authors:** Riccardo Geletti, Giuseppe Brancatelli, Edy Forlin, Nicolò Bertone, Anna Del Ben

**Affiliations:** a National Institute of Oceanography and Applied Geophysics – OGS, Borgo Grotta Gigante 42/C 34010, Sgonico, Trieste, Italy; b Department of Mathematics, Informatics and Geosciences, University of Trieste, Via Weiss, 1 34127, Trieste, Italy

**Keywords:** Seismic data processing, OCSS15 project, SISOC project, Carbonate platform margin, Adriatic continental margin, SEGY format

## Abstract

This dataset presents an integrated collection of multichannel seismic (MCS) profiles acquired along the southeastern Apulia continental margin (Otranto Channel, southern Adriatic Sea) during the OCSS15 (2015), SISOC (2010), and Mediterranean Sea (MS, 1971) geophysical surveys conducted by the Istituto Nazionale di Oceanografia e di Geofisica Sperimentale (OGS).

The dataset includes seismic lines acquired with different streamer lengths and energy sources, providing complementary resolution and penetration of the shallow and intermediate subsurface. High-resolution OCSS15 profiles (short streamer and Mini GI gun) image the upper sedimentary units, while SISOC data (longer streamer and higher-energy source) provide deeper subsurface constraints. The MS-29 profile (1971), recently reprocessed using Pre-Stack Depth Migration (PSDM), adds further deep structural information.

Standardised processing workflows were applied to all lines to enhance the signal-to-noise ratio, suppress multiples, and preserve relative amplitudes. The dataset is provided in SEGY format, including both stacked and migrated sections.

Given the rising costs and environmental restrictions associated with active marine seismic sources (e.g. air guns), new seismic acquisitions for scientific research are increasingly limited. Consequently, the reuse, reprocessing, and open dissemination of legacy and previously acquired datasets are becoming more important. This compilation offers a valuable multi-resolution geophysical resource for structural, stratigraphic, and fluid-flow studies, as well as geo-hazard assessment in the southern Adriatic Sea, supporting data accessibility and research reproducibility.

Specifications Table

**Table:** 

Subject	Earth and Environmental Sciences
Specific subject area	Marine Geology and Geophysics: Multichannel Seismic (MCS) data acquisition and processing from the Otranto Channel, southern Adriatic Sea.
Type of data	Processed multichannel seismic reflection seismic data, stacked and migrated profiles, in SEGY format.
Data collection	Data were acquired during the OCSS15 (7–12 April 2015) and SISOC (12–16 October 2010) geophysical surveys by the research vessel R/V OGS Explora. The OCSS15 dataset was collected using a 300 m digital streamer and a 0.98 L Mini GI gun, while the SISOC dataset used a 1500 m, 120-channel streamer and two GI guns (11 L). The MS29 profile was acquired by the ship ``Marsili'' in 1971 with a 2400 m streamer of 24 traces; the shot interval was 200 m, resulting in a fold coverage of 600 %. MCS data were processed using Aspen Technology® Echos and Geodepth software at the SEISLAB laboratory of OGS, with workflows including SRME/WEMA multiple attenuation, pre-stack time migration (PSTM), and pre-stack depth migration (PSDM) [1].
Data source location	Data were acquired by National Institute of Oceanography and Applied Geophysics (OGS) in the Otranto Channel area (Geographical coordinates: approximately 39°30′–40°00′ N, 18°30′–19°10′ E (Otranto Channel, southern Adriatic Sea). Raw data are stored at www.snap.ogs.it (OGS).
Data accessibility	Repository name: Mendeley Data Data identification number: 10.17632/xgv9nd49xg Direct URL to data: https://data.mendeley.com/datasets/xgv9nd49xg
Related research article	The dataset described here supports the analyses presented in [2].

## 1 Value of the Data

**Fig. 1:**
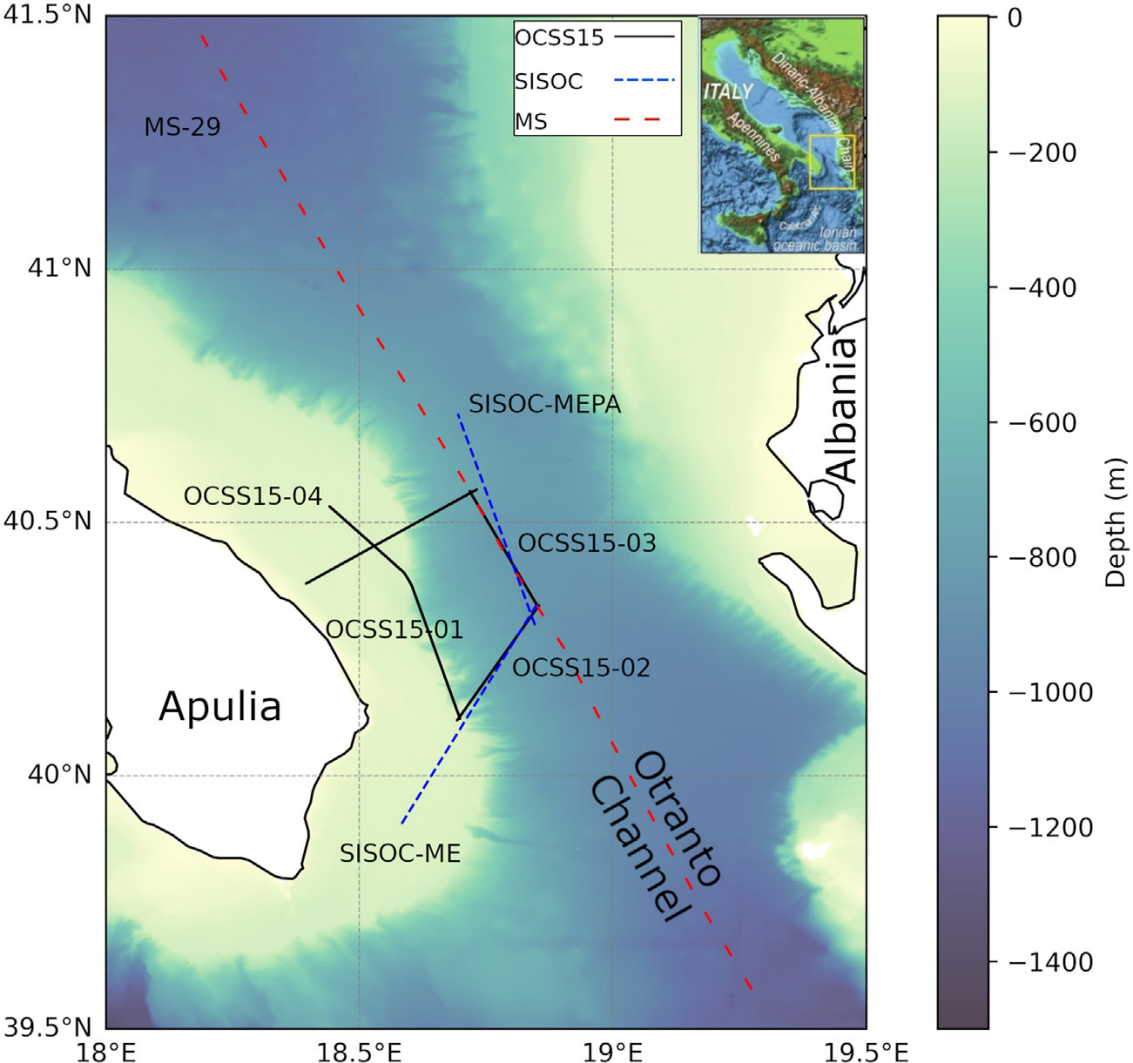
Position map on the bathymetry of the seismic lines.
• It allows identification of fault-controlled fluid migration features, including gas seepage indicators, as well as slope instability deposits such as mass-transport deposits, debris flows, and turbidity currents.
• The combination of OCSS15, SISOC, and MS datasets supports multi-scale analysis of margin architecture, enabling correlations between tectonic structures, sedimentary processes, and seafloor morphology at different resolutions.
• The availability of raw and processed SEG-Y data, along with navigation files and metadata, facilitates reuse for seismic interpretation, geological modelling, and advanced processing workflows, including seismic attribute analysis and data-driven approaches such as machine learning.

• This compilation offers a multi-resolution seismic dataset from the Otranto Channel, improving accessibility and reproducibility of existing data.
• The dataset integrates multichannel seismic (MCS) profiles from the south-eastern Apulia continental margin (Fig. 1), enabling detailed imaging of the Plio–Quaternary succession and the underlying carbonate platform.

## 2 Background

The Otranto Channel, located between the southeastern tip end of the Salento Peninsula and the Albanian coast, represents the main connection between the Adriatic and Ionian seas, with a minimum width of approximately 70 km. Its physiography is controlled by tectonic activity, sedimentary processes, and bottom-current dynamics affecting the continental margins.

The southeastern Apulian offshore includes the carbonate platform and the continental slope. The continental shelf is characterized by widespread erosion and sediment reworking, mainly associated with the passage of dense water masses and seasonal cascading events along the upper slope [3,4]. The continental slope is dissected by a network of straight gullies and submarine canyons that extend from the platform edge towards the basin.

The integration of the OCSS15, SISOC, and MS-29 datasets aims to document the detailed morphology and internal structure of this margin through high-resolution seismic imaging [5]. These data provide a geophysical framework that supports ongoing studies on the stratigraphic and structural evolution of the southern Adriatic region, enabling further multidisciplinary analyses of the tectono-sedimentary processes shaping the Otranto Channel [2].

## 3 Data Description

A key characteristic of this dataset is the integration of seismic profiles acquired with different acquisition geometries and resolutions, combining short-streamer high-resolution data with long-streamer deep-penetration datasets. This multi-scale configuration allows the analysis of shallow sedimentary features and deeper structural elements within a single framework. Furthermore, the inclusion of both legacy (MS-29) and modern datasets, all provided in SEGY format with consistent processing levels, makes this dataset suitable for comparative studies and advanced reprocessing applications.

The Otranto Channel dataset described in this article (Fig. 1) is publicly available in the designated online repository and can be accessed without restrictions. The dataset consists of three main geophysical data collections acquired in the southeastern Adriatic Sea (Table 1): (1) the OCSS15 multichannel seismic (MCS) dataset [6], (2) the SISOC MCS dataset [7], (3), and the MS-29 MCS line [1,8].

**Table 1:** Summary sheet of MCS lines from the Otranto Channel dataset, showing the shot point (SP) numbers (start of line SP number – Sol SP No.; end of line SP number – Eol SP No.) and the corresponding geographical and UTM 34 projected coordinates. The latter are recorded for each line in the trace headers in fixed-point format.

Line name	Shot Point	Lon. East	Lat. North	X(UTM34) (m)	Y(UTM34) (m)	Shot No.	Distance (m)
OCSS15–01	Sol 100	18°26′28″	40°31′54″	283,269	4,489,909	5676	51,496
	Eol 5775	18°41′55″	40°06′43″	303,871	4,442,714		
OCSS15–02	Sol 100	18°41′34″	40°06′34″	303,355	4,442,451	3085	28,698
	Eol 3184	18°51′16″	40°20′10″	317,745	4,467,280		
OCSS15–03	Sol 100	18°51′13″	40°19′53″	317,663	4,466,743	3011	28,060
	Eol 3110	18°43′04″	40°33′42″	306,800	4,492,615		
OCSS15–04	Sol 100	18°43′53″	40°33′54″	307,962	4,492,936	3781	35,228
	Eol 3880	18°23′42″	40°22′45″	278,857	4,473,089		
SISOC-ME	Sol 100	18°35′14″	39°54′40″	293,442	4,420,039	2159	53,606
	Eol 2258	18°51′09″	40°20′30″	317,600	4,467,893		
SISOC-MEPA	Sol 100	18°50′47″	40°17′52″	316,962	4,463,034	1032	47,999
	Eol 2031	18°41′40″	40°42′51″	305,256	4,509,584		
MS-29	Sol 1	19°16′27.8″	39°34′40.7″	351,795	4,382,340	1156	228,877
	Eol 1156	18°11′13.3″	41°27′52.6″	265,082	4,594,155		

The repository (doi.org/10.17632/xgv9nd49xg) is organised into dedicated folders for each survey, with subfolders containing processed seismic sections and navigation files. All files retain their original naming conventions, and the folder structure mirrors the organisation used in the public repository. This approach ensures transparency, facilitates data reuse, and supports reproducibility in future research.

## 4 Experimental Design, Materials and Methods

### 4.1 Acquisition geometry and survey parameters

**OCSS15 seismic lines:** The seismic source used during the OCSS15 survey consisted of a single 60 in^3^ (Table 2) mini-GI gun mounted on a 1 m steel beam and operated in harmonic mode (30 in^3^ Generator + 30 in^3^ Injector) to ensure a stable and high-quality signal while minimising energy loss [6]. The shot point spacing was 9.375 m, corresponding to a time interval of approximately 4.8 s at a vessel speed of 3.8 knots, allowing sufficient time for the compressor to recharge at a nominal pressure of 140 bar. The gun was towed at a depth of 1.5 m, producing a ghost notch of about 500 Hz, which defines the upper limit of the amplitude spectrum. Based on the dominant frequencies and the λ/4 Rayleigh criterion for seismic velocities between 1500 m/s and 3000 m/s, the expected vertical resolution ranges from approximately 1 m for shallow reflectors to 2 m for the deepest ones. The OCSS15 seismic data were acquired using a 96-channel digital streamer, 300 m long, with a channel spacing of 3.125 m and a shot interval of 9.375 m. The near offset, defined by the tow leader length, was set to 31.25 m. The streamer was towed at a depth of approximately 1.5 m below the sea level. To maintain a constant towing depth, four depth-control devices (“birds”) were used and operated through a TAP bird control system, which continuously monitored streamer depth and automatically adjusted the wings to maintain the target level.

**Table 2:** Summary sheet of MCS acquisition parameters.

MCS reflection profiles	OCSS15 (−01, −02, −03, −04)	SISOC (-ME, -MEPA)	MS-29
**Vessel**	R/V OGS-Explora	R/V OGS-Explora	R/V Marsili
**Time Period**	2015	2010	1971
**Source Type**	Mini GI-Gun (1 l)	GI-guns (11 l)	Flexotir (150 g of Dynamite)
**Recording Filters**	3 Hz LC Antialias HC	3–0.8xFn Hz	10–72 Hz
**Recording Length**	6 s	8 s	10 s
**Sampling Rate**	0.5 ms	1 ms	4 ms
**Group Interval**	3.125 m	12.5 m	100 m
**Shot Interval**	9.375 m	25 m	200 m
**Number of Groups**	96	120	24
**Near offset**	30 m	55 m	270 m
**Coverage**	1600 %	3000 %	600 %
**Streamer length**	300 m	1500 m	2400 m
**Streamer depth**	2 m	3 m	10 m
**Source depth**	2 m	3 m	14 m

**SISOC seismic lines:** This dataset was acquired using two GI guns configured in a near-harmonic operating mode (2 × 250 in^3^ Generator + 105 in^3^ Injector), providing an optimal balance between penetration and resolution [7]. The receiving system consisted of a 120-channel digital streamer, 1500 m long, with a group spacing of 12.5 m and a minimum offset of 25 m. For safety reasons, particularly due to vessel traffic, the streamer was towed at a depth of 4–4.5 m, while the gun array was maintained at a depth of 5 m to avoid entanglement and potential damage. Based on the combined ghost effect from these depths (assuming a sound velocity of 1500 m/s), the effective upper frequency limit was approximately 150 Hz. According to the λ/4 Rayleigh criterion and assuming an average velocity of 2000 m/s, the minimum resolvable bed thickness was estimated at around 3–4 m. The shot interval was set to 25 m, allowing for high fold coverage (up to 3000 %), while maintaining a minimum offset of 55 m depending on sea conditions. The fixed receiver group spacing ensured consistent horizontal sampling of 12.5 m in the stacked section, providing high-quality imaging of the seafloor and shallow subsurface structures.

**MS seismic lines:** The MS (Mediterranean Sea Project) dataset was a pioneering seismic exploration programme carried out between 1969 and 1982 by the Istituto Nazionale di Oceanografia e di Geofisica Sperimentale (OGS) of Trieste, using the CNR research vessel Marsili [8]. Approximately 28,000 km of seismic lines were acquired across the Mediterranean Sea, representing the first regional-scale application of multifold seismic reflection techniques for scientific research.

The MS-29 line was acquired using a 2400 m long multichannel streamer (towed at a mean depth of 25 m) and synchronised Flexotir seismic sources, with a shot spacing of 200 m, providing fold coverage of up to 600 %. This configuration enabled imaging of both shallow sedimentary units and deeper crustal structures.

**The** Table 2 summarises the main acquisition parameters of the multichannel seismic (MCS) profiles presented in this paper. The table outlines key technical details such as source configuration, streamer length, shot and group intervals, and towing depths, which together determine the overall resolution and penetration capability of the seismic data.

### 4.2 Processing sequence and methodologies

The processing of the seismic lines by the Echos and GeoDepth software from Aspen Technology at the SEISLAB laboratory of OGS aimed at attenuating the short and long-period multiples, preserving relative amplitude, increasing the signal to noise ratio, and improving seismic imaging [9].

The spectral central frequency of the three datasets (MS-29, SISOC and OCSS15) are 35, 70 and 100 Hz respectively, corresponding to low, medium and high resolution. Moreover, different acquisition configurations affect the choice of the appropriate processing sequence. In particular, length and maximum offset constrain velocity analysis and the maximum depth of reliable velocity estimation As the accuracy and maximum depth of the velocity field influenced the imaging strategy, the final section was a post-stack time-migrated section for the OCSS15 dataset, a pre-stack time-migrated section for the SISOC dataset, and a pre-stack depth-migrated section for the MS-29 line.

**Fig. 2:**
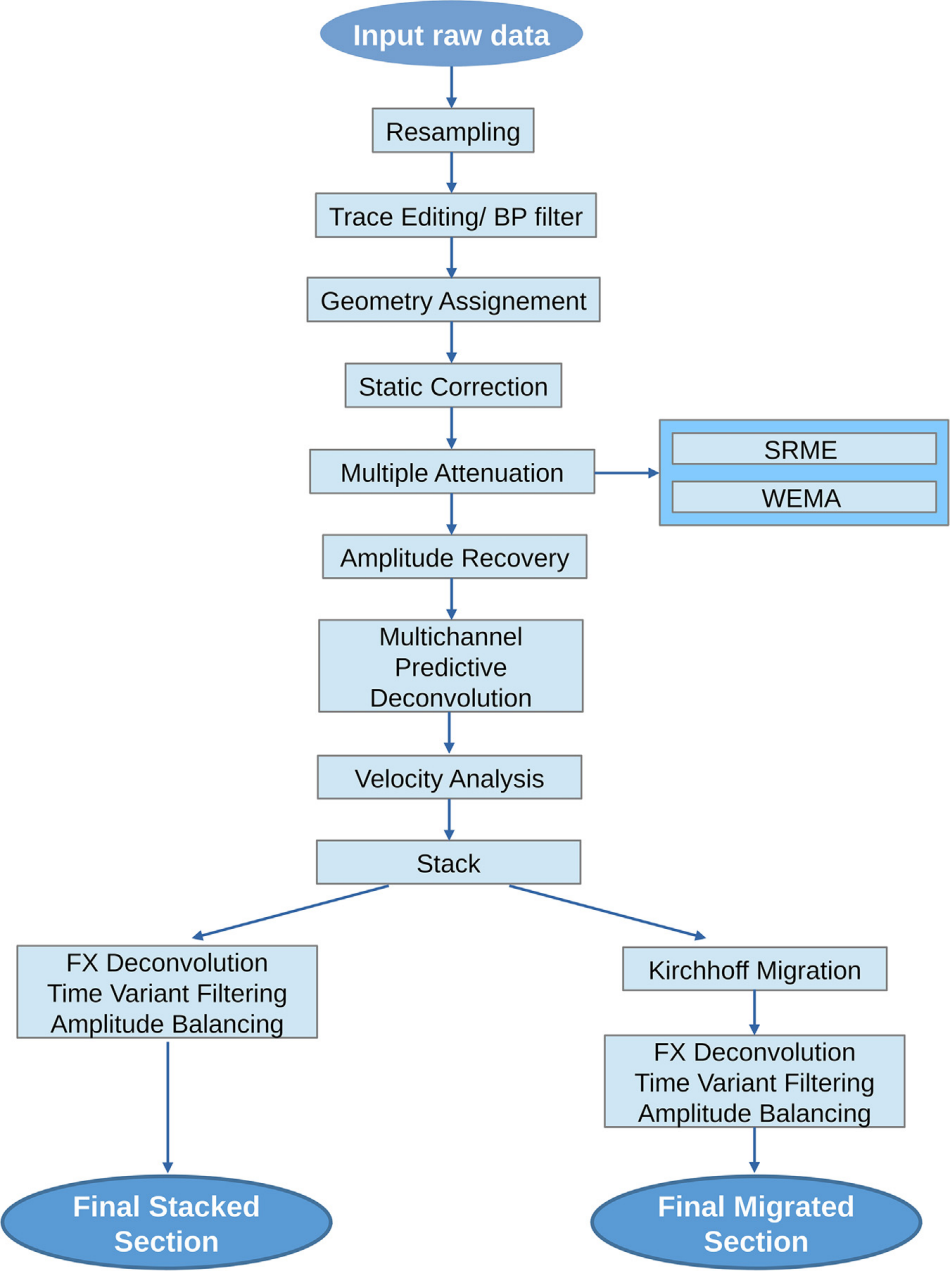
Flowchart of the processing steps for the OCSS15 dataset.

**OCSS15 seismic lines:** The processing sequence of this dataset, described in Fig. 2, includes resampling from 0.5 to 1 ms (with a Nyquist frequency of 500 Hz), a bandpass filtering (5–10–250–500 Hz, to eliminate swell noise), geometry assignment, multiple attenuation, amplitude recovery (by spherical divergence correction), multichannel predictive deconvolution, velocity analysis, and stacking of common-midpoint (CMP) traces. A post-stack Kirchhoff migration was applied using the stack velocity field scaled to 90 %. Finally, fx-deconvolution was applied to increase the lateral continuity of the reflectors, time-varying filtering to compensate for the loss of frequencies with depth, and trace amplitude balancing.

**Fig. 3:**
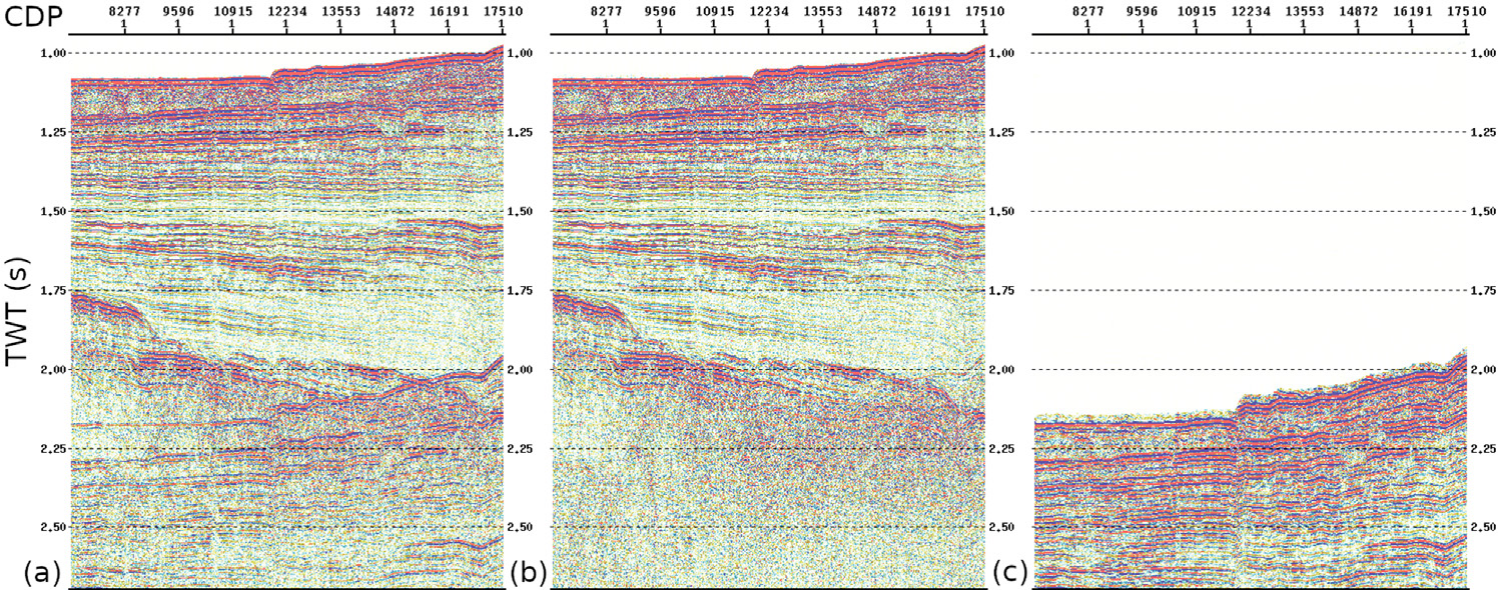
Segment of the OCSS15–03 seismic profile showing successive processing stages: (a) section before multiple attenuation, (b) section after application of the SRME/WEMA multiple removal method, and (c) difference section representing the removed multiples.

One of the main challenges affecting the OCSS15 seismic dataset is the presence of multiple reflections, particularly in the continental shelf area. These coherent noise effects were attenuated using the Surface-Related Multiple Elimination/Wave Equation Multiple Attenuation (SRME/WEMA) method, according to the workflow adopted by [1] for the MS-29 line, which was subsequently included in the Otranto Channel dataset presented in this paper. An example of the processing results is shown in Fig. 3, where multiple attenuation was applied to a segment of the OCSS15–03 profile. The panel on the left (a) shows the section before the application of SRME/WEMA, the centre panel (b) shows the section after the application, and the panel on the right (c) shows the multiples removed from the data.

The temporal resolution was improved by applying multichannel predictive deconvolution with a gap length of 12 ms and an operator length of 50 ms. These parameters were selected by analysing the autocorrelation of the data traces. After sorting the data into CMP gathers, classical velocity analysis was performed to determine stacking velocities, which were then used for the normal move-out (NMO) correction. A mute function was designed and applied to the data to prevent wavelet stretching, particularly at far offsets and shallow depths, after which the CMP gathers were stacked. As the fold coverage is 1600 %, the stacked section achieved a fourfold increase in the signal-to-noise ratio. To collapse diffraction and recover the correct geometries of the reflectors, we applied post-stack Kirchhoff migration. Post-stack processing includes incoherent noise attenuation in the frequency-space domain (f-x deconvolution). After time-frequency analysis, a time-variant bandpass filter was designed for three windows, following the water bottom profile (WB time) (Table 3).

**Table 3:** Time-variant filtering for OCSS15 and SISOC lines. The time windows indicate the start and end times of the filter application, based on the water bottom time (WB). Frequency values represent the low frequency, cut-off low frequency, cut-off high frequency, and high frequency of the trapezoidal bandpass filter, respectively.

OCSS15	Time window start (ms)	Time window end (ms)	Bandpass filtering frequencies (Hz)
	WB	WB+400	20/40–120/240
	WB+800	WB+1200	15/30–100/200
	WB+1400	3000	10/20–80/160
**SISOC**	WB	WB+500	15/30–100/200
	WB+1000	WB+1600	10/20–80/160
	WB+2000	5000	5/10–60/120

**Fig. 4:**
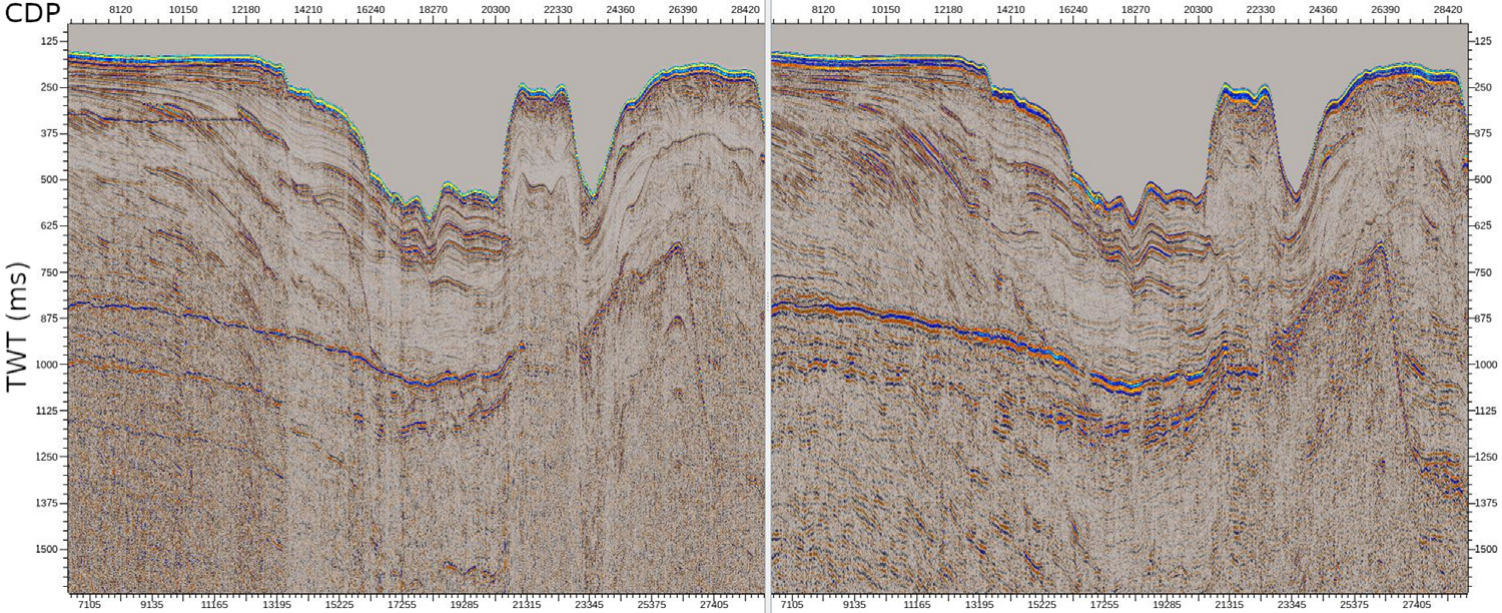
A section of the OCSS15–01 line before (left) and after (right) the complete processing sequence was applied. Coherent (multiples) and incoherent noise were attenuated and temporal and spatial resolution were impèroved.

The final result is a high-resolution image of the geological structures in the shallow part of the Earth's crust, down to approximately 2.5 s TWT (Fig. 4).

**Fig. 5:**
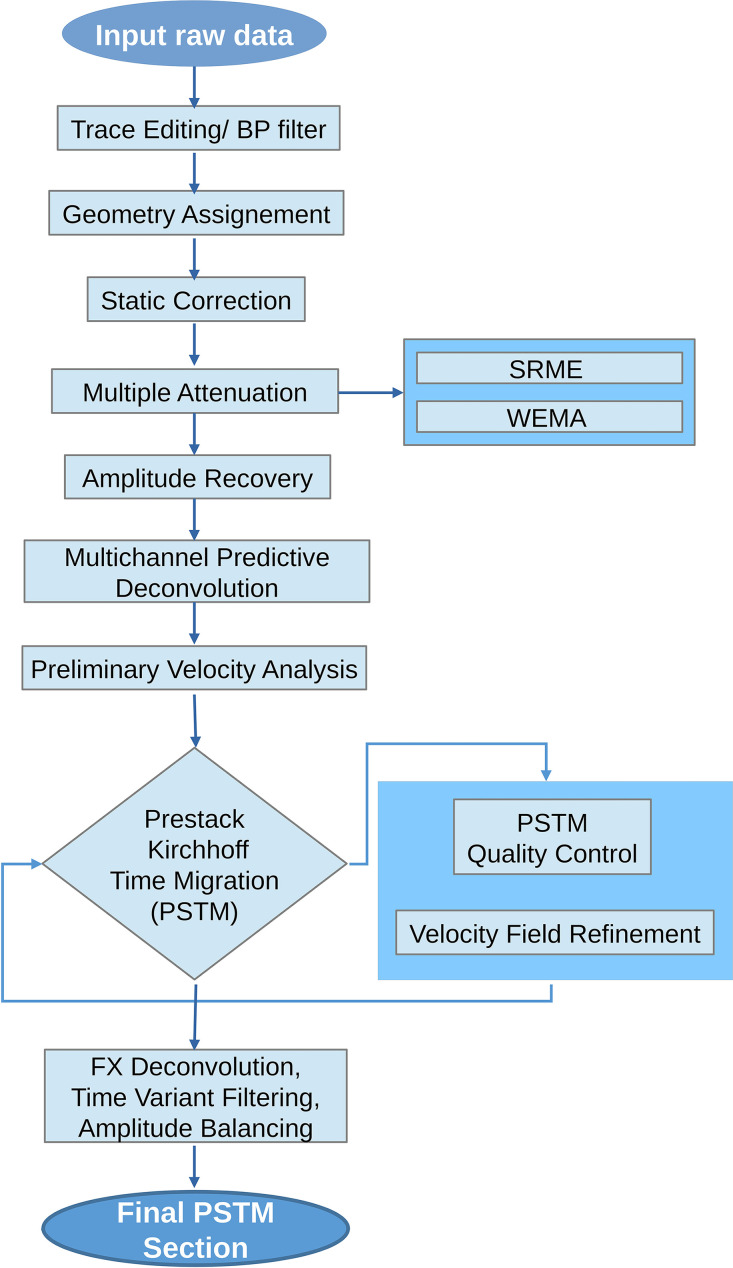
Flowchart of the processing steps for the SISOC dataset.

**SISOC seismic lines:** The main difference in the processing sequence between the SISOC lines and the OCSS15 lines is the migration strategy used (Fig. 5).

As the SISOC lines were acquired with a streamer length of 1500 m, accurate velocity information could be inferred at greater depths in the recorded data and thus apply pre-stack time migration (PSTM), which is more sensitive to velocity variations. After an initial preliminary velocity analysis, PSTM was applied and a detailed velocity analysis was performed every 120 common image gathers (CIGs) [1,9], corresponding to 1500 m. The PSTM and velocity analysis refinement were applied iteratively until a low residual move-out error was achieved, indicating flat reflections on the CIGs [1]. The final time-variant filtering differs from the OCSS15 dataset because the SISOC data have higher penetration and lower resolution (Table 3).

**MS seismic lines:** The complete processing of MS-29 seismic line, that includes velocity modelling through reflection tomography and depth imaging, is described in [1].

**Fig. 6:**
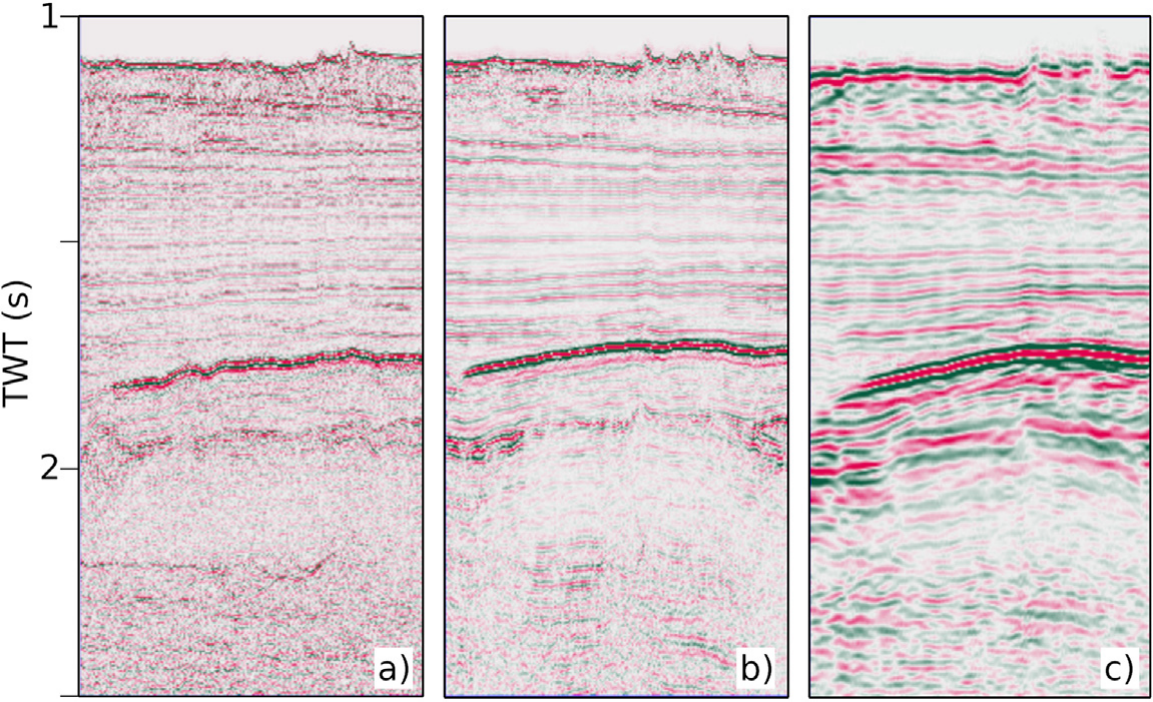
Seismic images: comparison of sections from the OCSS15–03 (a), SISOC-MEPA (b), and MS-29 (c) lines, acquired approximately along the same path (see the maps in Fig. 1), showing different seismic resolutions.

**Comparison of seismic resolution and penetration depth across datasets:** The seismic images from the different datasets, acquired approximately along the same path, depict the geological stratigraphy of the Otranto Channel area at varying resolutions (Fig. 6). Comparing the three images, the resolution decreases from a) to c), allowing greater signal penetration into deeper geological layers.

The processing of the new lines aimed to attenuate short- and long-period multiples, preserve relative amplitudes, increase the signal-to-noise ratio, and improve overall seismic imaging. These results proved particularly valuable, enabling a meaningful partial reinterpretation of the vintage, lower-resolution profiles.

## Limitations

The dataset has some limitations related to acquisition geometry, resolution, and data consistency across surveys.

The OCSS15 seismic lines, acquired with a short streamer (300 m), offer limited offset coverage, which constrains velocity analysis and reduces the reliability of deeper structural imaging compared to longer-streamer datasets such as SISOC and MS-29.

Differences in acquisition parameters among the OCSS15, SISOC, and MS datasets (including streamer length, source energy, and fold coverage) result in variable resolution and penetration depth, which may affect direct comparability between profiles. This could also be considered a strength, as each dataset contributes to analysing characteristics of different magnitude. Their integration provides a more comprehensive description of the study area.

Additionally, coherent noise from multiple reflections is present, particularly in shallow shelf areas, although it has been attenuated through dedicated processing techniques such as SRME and WEMA, as described in the previous chapter.

These limitations also present opportunities for further work, including the application of advanced reprocessing workflows, improved velocity modelling, and multi-dataset integration approaches aimed at enhancing seismic imaging and interpretation.

## Ethics Statement

The authors read and follow the ethical requirements for publication in Data in Brief. This work does not involve the use of human subjects, animal experiment and data collected from social media platforms.

## Credit Author Statement

**R. Geletti:** Conceptualization, Data curation, Formal analysis, Funding acquisition, Investigation, Methodology, Resources, Supervision, Validation, Visualization, Writing – original draft, Writing – review & editing. **G. Brancatelli:** Conceptualization, Data curation, Formal analysis, Methodology, Resources, Validation, Visualization, Writing – original draft, Writing – review & editing. **E. Forlin:** Data curation, Formal analysis, Methodology, Resources, Validation, Writing – review & editing. **N. Bertone:** Data curation, Formal analysis, Methodology. **A. Del Ben:** Conceptualization, Investigation, Supervision, Validation, Visualization, Writing – review & editing. **A. Mocnik:** Methodology. **L. Petronio:** Conceptualization, Funding acquisition, Investigation, Methodology.

## Data Availability

Mendeley DataSEG-Y Integrated Multichannel Seismic Profiler Dataset from the Southeastern Apulia Continental Margin (Otranto Channel, Southern Adriatic Sea) (Original data)
